# Modeling mammalian spermatogonial differentiation and meiotic initiation *in vitro*

**DOI:** 10.1242/dev.200713

**Published:** 2022-11-16

**Authors:** Oleksandr Kirsanov, Taylor Johnson, Taylor Malachowski, Bryan A. Niedenberger, Emma A. Gilbert, Debajit Bhowmick, P. Hande Ozdinler, Douglas A. Gray, Kelsey Fisher-Wellman, Brian P. Hermann, Christopher B. Geyer

**Affiliations:** ^1^ Department of Anatomy and Cell Biology, Brody School of Medicine, East Carolina University, Greenville, NC 27834, USA; ^2^ Flow Cytometry Facility, Brody School of Medicine, East Carolina University, Greenville, NC 27834, USA; ^3^ Department of Neurology, Feinberg School of Medicine, Northwestern University, Evanston, IL 60611, USA; ^4^ Department of Biochemistry, Microbiology, and Immunology, University of Ottawa, Ottawa, K1H 8M5, Canada; ^5^ Cancer Therapeutics, Ottawa Hospital Research Institute, Ottawa, K1H 8L6, Canada; ^6^ Department of Physiology, Brody School of Medicine, East Carolina University, Greenville, NC 27858, USA; ^7^ East Carolina Diabetes and Obesity Institute, East Carolina University, Greenville, NC 27858, USA; ^8^ Department of Neuroscience, Developmental and Regenerative Biology, University of Texas at San Antonio, San Antonio, TX 78249, USA

**Keywords:** Testis, Spermatogenesis, Meiosis, Spermatogonia, Spermatocytes

## Abstract

In mammalian testes, premeiotic spermatogonia respond to retinoic acid by completing an essential lengthy differentiation program before initiating meiosis. The molecular and cellular changes directing these developmental processes remain largely undefined. This wide gap in knowledge is due to two unresolved technical challenges: (1) lack of robust and reliable *in vitro* models to study differentiation and meiotic initiation; and (2) lack of methods to isolate large and pure populations of male germ cells at each stage of differentiation and at meiotic initiation. Here, we report a facile *in vitro* differentiation and meiotic initiation system that can be readily manipulated, including the use of chemical agents that cannot be safely administered to live animals. In addition, we present a transgenic mouse model enabling fluorescence-activated cell sorting-based isolation of millions of spermatogonia at specific developmental stages as well as meiotic spermatocytes.

## Teaser

**Summary:** Here, we report robust and reliable tools to model mammalian meiotic initiation *in vitro* and isolate pure populations of millions of cells undergoing the mitosis-to-meiosis transition for biochemical assays.

## INTRODUCTION

The daily production of millions of sperm throughout the mammalian male reproductive lifespan occurs during spermatogenesis, a developmental system based on the tissue-specific stem cell activities of spermatogonia (Russell, 1993). Spermatogonia exist as three subtypes: stem [spermatogonial stem cell (SSC)], undifferentiated progenitor and differentiating. SSCs provide the foundation for spermatogenesis and undergo asymmetric cell divisions – their progeny either remain a stem cell (self-renewal) or become transit-amplifying undifferentiated progenitor spermatogonia (de Rooij, 2001) that proliferate before committing to differentiation in response to retinoic acid (RA) (Busada et al., 2015; Busada and Geyer, 2016; Griswold, 2016). Once differentiation is initiated, newly formed type A_1_ spermatogonia undergo five cell divisions [forming types A_2-4_, intermediate (In) and B], which together last ∼8 days in mice (∼16 days in human) prior to entering meiosis as preleptotene spermatocytes (Clermont, 1972; Hilscher and Hilscher, 1976; Oakberg, 1956a,b; Roosen-Runge, 1952).

Multiple groups have focused on defining the molecular mechanisms and signaling pathways required for SSC self-renewal (Kanatsu-Shinohara and Shinohara, 2013; Phillips et al., 2010; Yang and Oatley, 2014; Yoshida, 2012). A significant part of this progress has been made using culture-adapted spermatogonia, in which undifferentiated spermatogonia (typically, only a small percentage of which are SSCs) are maintained on a layer of irradiated mouse embryonic fibroblasts for months in the presence of factors that support the undifferentiated fate, including GDNF and bFGF (also known as FGF2) (Kubota et al., 2004a,b). The utility of this long-term culture system to study SSC biology has been confirmed by the successful transplantation of cultured spermatogonia into testes of recipient male mice lacking a germline (Kubota et al., 2004a,b; Nagano, 2003). In contrast, the ∼8 day-long spermatogonial differentiation program has received considerably less attention. As a consequence, the molecular mechanisms and cellular pathways directing this essential developmental program that prepares male germ cells for meiotic initiation remain largely undefined.

There are two key technical barriers that underlie why so little is currently known about differentiation and meiotic initiation. The first barrier has been the lack of a robust, straightforward and reliable *in vitro* developmental system that begins with mitotic spermatogonia and culminates with meiotic spermatocytes. Such a system would enable a variety of interventions (e.g. agonists/antagonists, drug screening) to dissect the mechanisms driving spermatogonia differentiation and meiotic initiation. The second barrier has been our field's inability to readily isolate highly enriched populations of millions of spermatogonia at each stage of their development or preleptotene spermatocytes for biochemical studies. Historically, cell sedimentation at unit gravity through a differential bovine serum albumin (BSA) gradient (termed StaPut) was used to isolate male germ cells (Bellve et al., 1977; Bryant et al., 2013; Romrell et al., 1976). However, this time-consuming method requires considerable expertise and specialized equipment, and cell diameters of the germ cell type to be isolated must significantly differ from those of other germ and somatic cell types. Although these significant size differences certainly exist for meiotic pachytene spermatocytes and postmeiotic haploid spermatids, they do not exist for progenitor or differentiating spermatogonia. Therefore, spermatogonia have been isolated using other methods, including magnetic-activated cell sorting (MACS) (van der Wee et al., 2001). This method relies upon antibody-based cell capture of specific cell-surface proteins, such as THY1 (Kubota et al., 2004a). Many studies have utilized THY1 as this surface marker in progenitor spermatogonia, although THY1 is also expressed in interstitial somatic cells, thus introducing potential significant contamination of spermatogonia cultures (Oatley et al., 2009). In addition, MACS-based approaches yield insufficient cell numbers for many downstream biochemical analyses. Owing to these limitations, flow cytometry has more recently been used to sort and isolate spermatogonia based on antibody capture of cell surface markers (Cai et al., 2020) or epifluorescence (Chan et al., 2014; Gewiss et al., 2021; Romer et al., 2018). Although fluorescence-activated cell sorting (FACS) has tremendous potential for reliable isolation of large numbers of spermatogonia, the majority of models in the literature come with limitations, including expression of fluorophores in only a subset of spermatogonia (e.g. SSCs and early progenitors during the first wave of spermatogenesis; Chan et al., 2014; Helsel et al., 2017) or infrequency of genetically appropriate male mice (thus relatively low cell yields/litter; Romer et al., 2018).

The models presented here overcome these two significant technical barriers by providing a robust set of tools for isolation of large numbers of spermatogonia at each stage of their development and employ a simplified *in vitro* model system to investigate the molecular and cellular changes during differentiation that prepare spermatogonia for meiosis.

## RESULTS

### Spermatogonial differentiation and meiotic initiation occur in the same time frame *in vivo* and *ex vivo*

**Fig. 1.**
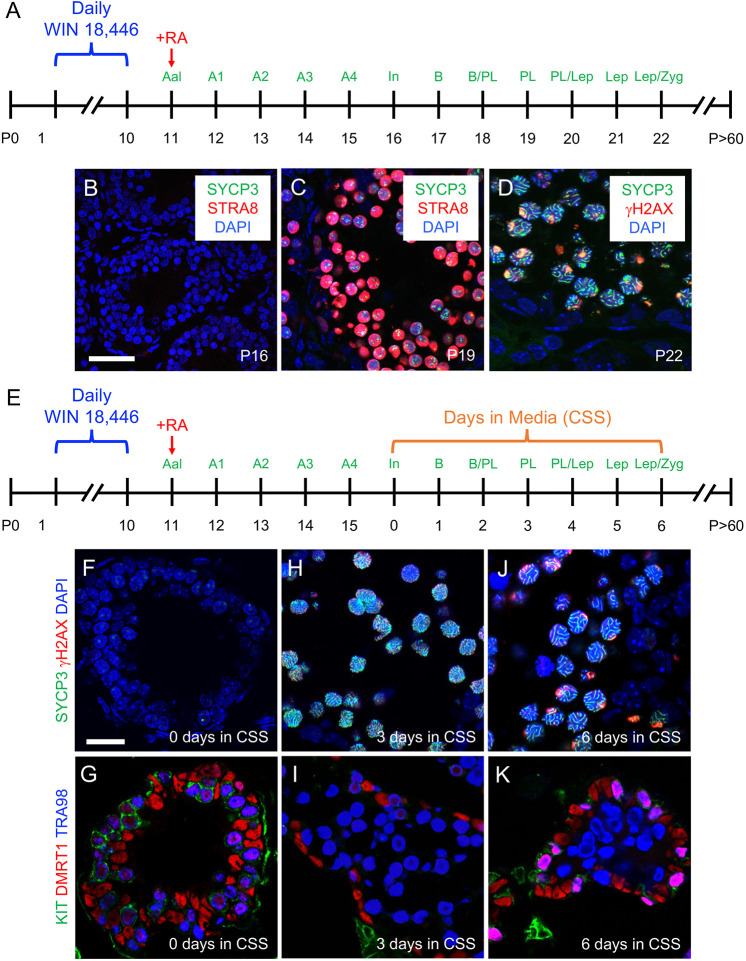
**Modeling spermatogonial differentiation and meiotic initiation *in vivo* and *ex vivo*.** (A) Timeline for synchronizing spermatogenesis *in vivo*. (B-D) Immunostaining for protein cell fate markers at postnatal day (P)16 (B), P19 (C) and P22 (D); the colors representing markers are indicated on each image. (E) Timeline for synchronizing spermatogenesis *in vivo* prior to harvesting tissue at P16 (day 0 of culture) for *ex vivo* cultures. (F-K) Immunostaining for protein fate markers at culture day 0 (F,G), 3 (H,I) and 6 (J,K); the colors representing markers are indicated to the left of each image. Scale bars: 50 µm (B), 25 µm (F). Each experiment for both *in vivo* and *ex vivo* synchronization was repeated five times (*n*=5) as separate biological replicates. Three (*n*=3) technical replicates were performed for each biological replicate. Each representative immunostaining was repeated three times. Aal, type A aligned undifferentiated; A1, type A1 differentiating; A2, type A2 differentiating; A3, type A3 differentiating; A4, type A4 differentiating; B, type B differentiating; CSS, charcoal-stripped serum; In, type intermediate differentiating; Lep, leptotene; PL, preleptotene; RA, retinoic acid; Zyg, zygotene.

To define the progression of spermatogonial differentiation and meiotic initiation *in vivo*, we modified a model of synchronized steady-state spermatogenesis (Fig. 1A) (Hogarth et al., 2013; Romer et al., 2018). Spermatogenesis was synchronized first by feeding mice the potent and selective RA synthesis inhibitor WIN 18,446 (Amory et al., 2011; Hogarth et al., 2011) from postnatal day (P)1 to P10, resulting in testes containing only STRA8-undifferentiated type A spermatogonia (Fig. S1A). At P11, mice were given a single dose of exogenous RA to initiate differentiation; as a result, spermatogonia were STRA8^+^ type A_1_ within 24 h (Fig. S1B). During the course of these experiments, we made the curious observation that, in testes from WIN 18,446-treated mice aged P8-10, a small but consistent population of STRA8^−^ spermatogonia always became preleptotene spermatocytes at P8 and appeared to enter meiosis as SYCP3^+^ spermatocytes (Fig. S2). These germ cells appear to be preprogrammed to differentiate and enter meiosis in RA-deficient testes and may represent the pool of differentiating spermatogonia predicted to directly emerge from precursor prospermatogonia at ∼P3 without first becoming stem or progenitor spermatogonia (Yoshida et al., 2006). These spermatocytes disappeared from the testis by P11. Then, owing to the precise timing of spermatogenesis, advanced germ cell types predictably appeared on subsequent days – type In STRA8^−^/SYCP3^−^ differentiating spermatogonia appeared on P16 (Fig. 1B) and STRA8^+^/SYCP3^punctate^ preleptotene spermatocytes undergoing meiosis appeared by P19 (Fig. 1C). By P22, spermatocytes were SYCP3^ribbons^/γH2AX^punctate^ and in leptonema/zygonema of meiosis (Fig. 1D), and by P30 had formed normal-appearing haploid spermatids (Fig. S1C). At P50, sperm were present in the cauda epididymides (Fig. S1D).

We next modeled spermatogonial differentiation and meiotic initiation in an *ex vivo* approach using testis explants from mice with synchronized spermatogenesis. Testes from P16 mice (containing type In spermatogonia as the most advanced germ cell type) were cut into small pieces and maintained in hanging drops in media containing charcoal-stripped serum (CSS) (Fig. 1E). These explants retained the testis architecture surrounding spermatogonia and, at the initiation of culture, contained SYCP3^−^/γH2AX^−^/KIT^+^/DMRT1^+^ differentiating spermatogonia as expected (Fig. 1F,G). After 3 days in culture, male germ cells were SYCP3^ribbons^/γH2AX^dim,punctate^/KIT^−^/DMRT1^−^, revealing that they had entered meiosis. Three days later, these meiotic spermatocytes were SYCP3^ribbons^/γH2AX^strong,punctate^/KIT^−^/DMRT1^−^ and had progressed into zygonema/pachynema (Fig. 1J,K). Overall, these explant cultures closely recapitulated the *in vivo* timeline (Fig. 1A-D).

### Recapitulating spermatogonial differentiation and meiotic initiation *in vitro*

Recently, it was reported that spermatogonia maintained in long-term *in vitro* cultures from developing testes either died (Sinha et al., 2021) or could only initiate meiosis under nutrient-restricted conditions (Zhang et al., 2021). Because spermatogonia differentiated and initiated meiosis in our *ex vivo* explant cultures in nutrient-rich media (Fig. 1E-K), we examined whether these events also occurred *in vitro* under similar conditions. First, from P5 mice, testes were harvested and enzymatically digested into single-cell suspensions that were placed into culture media containing CSS (Fig. S3A). At P6, testes contained only SYCP3^−^ differentiating spermatogonia as the most advanced germ cell type (Fig. S3B). By days 7 and 9 in culture, male germ cells were closely associated with (either adjacent to or on top of) clusters of DMRT1^+^ Sertoli cells, which often spontaneously formed ring structures (Fig. S3C). These DMRT1^−^/SYCP3^ribbons^ spermatocytes had clearly advanced, by day 9, into zygonema/pachynema of meiosis (Fig. S3D). Thus, neonatal spermatogonia were capable of entering and proceeding into meiosis *in vitro* in nutrient-rich media.

**Fig. 2.**
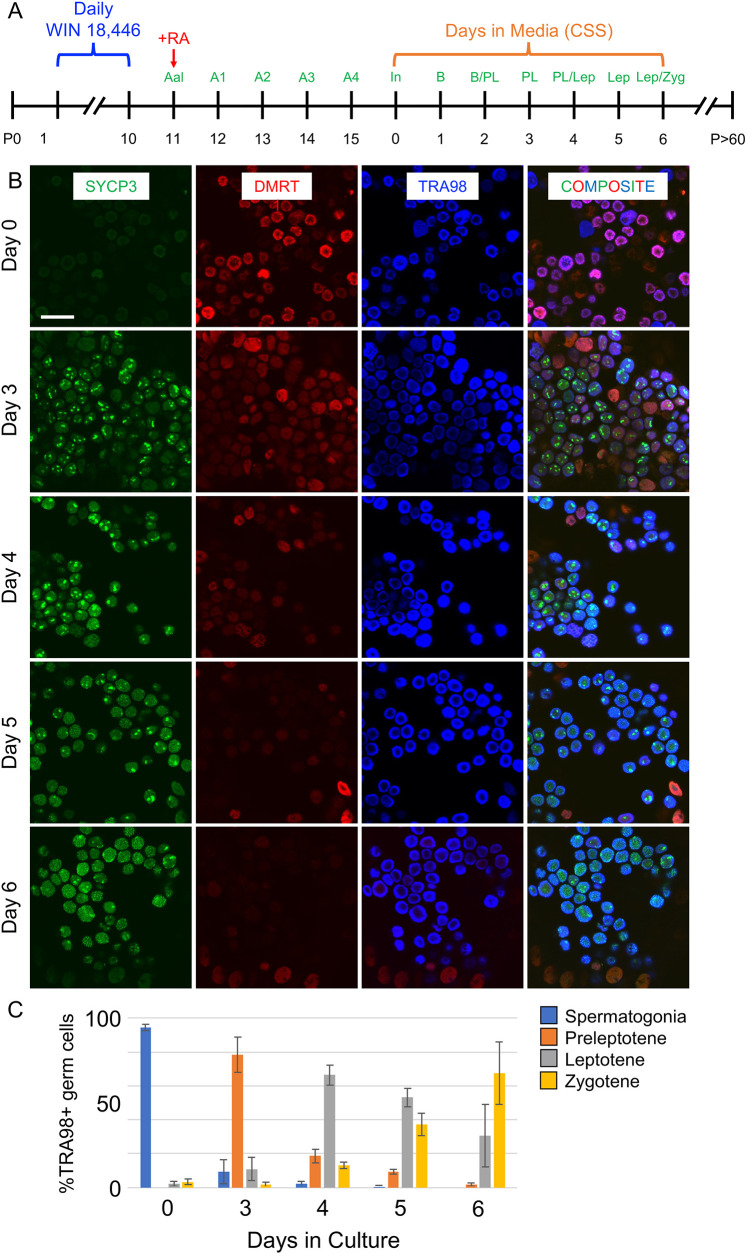
**Modeling spermatogonial differentiation and meiotic initiation *in vitro*.** (A) Timeline for synchronizing spermatogenesis *in vivo* prior to harvesting cells for *in vitro* cultures. (B) Immunostaining for protein fate markers at successive days of culture (indicated to the left of each row); the colors representing markers are indicated at the top of each column. (C) Quantitation of each germ cell type (premeiotic spermatogonia and preleptotene, leptotene and zygotene spermatocytes) is shown as a percentage of the entire germ cell population for each day of culture. Scale bar: 50 µm. The experiment was repeated five times (*n*=5) as separate biological replicates. Three technical replicates (*n*=3) were done for each biological replicate. Graphs represent mean±s.d. and error bars represent one s.d.

We next began *in vitro* cultures from spermatogonia undergoing SSC-derived steady-state spermatogenesis. Spermatogenesis was synchronized (Fig. 2A), and testes from P16 mice were enzymatically digested into single-cell suspensions, which were cultured in media containing CSS. As expected, the most advanced germ cells at P16 (day 0 of culture) were SYCP3^−^/DMRT1^+^ type In differentiating spermatogonia (Fig. 2B). By 3 days later, these spermatogonia had become SYCP3^punctate^/DMRT1^dim^ preleptotene spermatocytes, and by day 6 of culture they were SYCP3^ribbons^/DMRT1^−^ spermatocytes in zygonema/pachynema (Fig. 2B). Quantitation is provided in Fig. 2C. Therefore, using the same conceptual approach as in Fig. 1, spermatogonia *in vitro* completed differentiation and initiated meiosis in nutrient-rich conditions on the expected ∼8-day steady-state spermatogenesis time frame, replicating both *in vivo* and *ex vivo* observations.

### Identifying a mouse model with enhanced green fluorescent protein (EGFP) in spermatogonia and Sertoli cells undergoing steady-state spermatogenesis

**Fig. 3.**
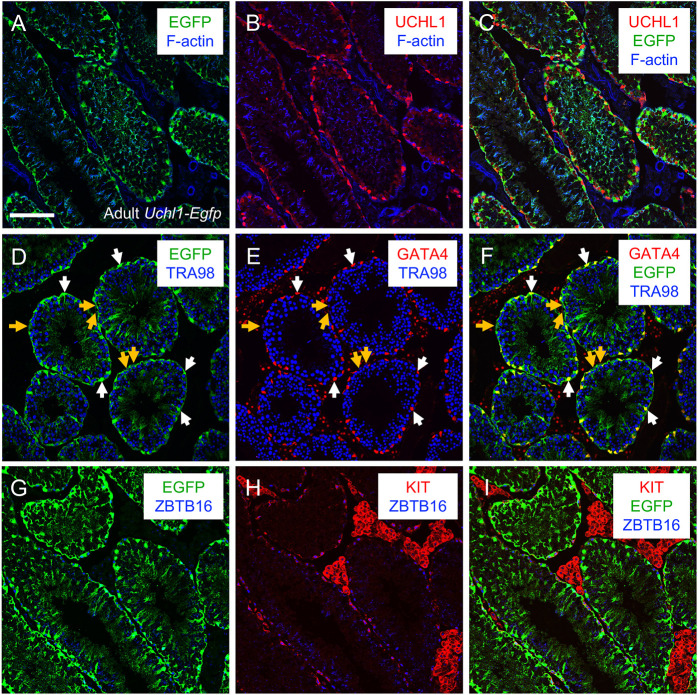
**UCHL1-EGFP is expressed in both spermatogonia and Sertoli cells in adult testes.** Immunostaining was done on adult (>P60) testes. (A-C) Immunostaining was done to colocalize UCHL1 (red) with EGFP (green), and F-actin was visualized using phalloidin (blue). (D-F) GATA4^+^ Sertoli cells (red) expressed EGFP, and all germ cells are marked with TRA98 (blue). White arrows point to Sertoli cells; yellow arrows point to germ cells. (G-I) EGFP was colocalized with spermatogonia that are undifferentiated (ZBTB16^+^, blue) or differentiating (KIT^+^, red). Scale bar: 50 µm. Each representative immunostaining was repeated three times (*n*=3).

We next sought to identify a transgenic mouse model with robust fluorophore expression in all spermatogonia, which would enable FACS-based isolation of spermatogonia for a variety of *in vitro* experimental approaches. We examined existing transgenic mouse models created using promoters directing tissue-specific gene expression in both brain and testes. Using this concept, we focused on ubiquitin carboxy-terminal hydrolase L1 (*Uchl1*; also known as PGP9.5; Edwards et al., 1991), a gene encoding a deubiquitinating enzyme with ligase and hydrolase activities (Larsen et al., 1996). *Uchl1* is highly expressed in sensory and sympathetic neurons (Calzada et al., 1994; Kent and Clarke, 1991), retinas (Esteve-Rudd et al., 2010) and male germ cells (Fujihara et al., 2011; Lee et al., 2016; Luo et al., 2006) in multiple species. In mice, UCHL1 protein is solely detectable in spermatogonia in developing testes, and in both spermatogonia and Sertoli cells in adult testes (Kwon et al., 2004). Transgenic mice were generated with the *Uchl1* gene promoter directing expression of *eGfp* to study motor neurons (Yasvoina et al., 2013), although the authors noted that both spermatogonia and Sertoli cells of P30 *Uchl1-eGfp* mice were EGFP^+^ (Genç et al., 2015). We first verified these results in adult (>P60) *Uchl1-eGfp* testes. Immunostaining revealed that 100% of GATA4^+^ Sertoli cells were also EGFP^+^ (Fig. 3A-F). Testes from *Uchl1* knockout (KO) mice were used as a negative control, revealing the specificity of anti-UCHL1 (Fig. S4A-C). To define which spermatogonia were EGFP^+^, we immunostained adult testes for the pan germ cell marker TRA98 (also known as GCNA; Enders and May, 1994) along with two established protein markers of spermatogonia fate – undifferentiated [zinc finger and BTB domain containing 16 (ZBTB16; also termed PLZF; Buaas et al., 2004; Costoya et al., 2004)] and differentiating [KIT proto-oncogene receptor tyrosine kinase (KIT; also termed c-KIT; Kissel et al., 2000; Manova et al., 1990; Schrans-Stassen et al., 1999; Sorrentino et al., 1991; Yoshinaga et al., 1991)] (Fig. 3G-I). All ZBTB16^+^ and KIT^+^ spermatogonia were EGFP^+^, consistent with previous reports revealing that both undifferentiated and differentiating spermatogonia express UCHL1 protein (Kon et al., 1999).

### All spermatogonia in the developing testis are EGFP^+^; Sertoli cells become EGFP^+^ at P17

**Fig. 4.**
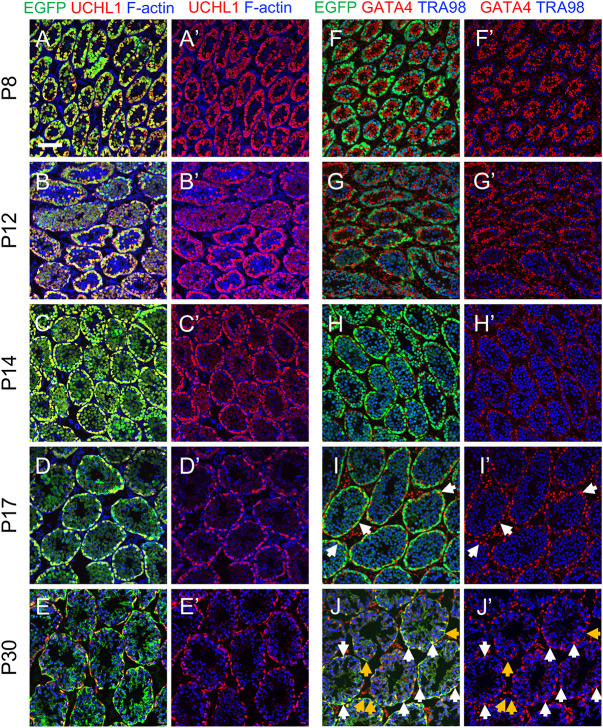
**UCHL1-EGFP expression is restricted to spermatogonia until P14.** (A-J′) Immunostaining for protein cell fate markers (indicated, with colors, at the top of each column) for mouse testes during the first wave of spermatogenesis. Ages are shown on the left of each row. White arrows point to Sertoli cells; yellow arrows point to germ cells. Scale bar: 50 μm. Each representative immunostaining was repeated three times (*n*=3).

We next examined the ontogeny of EGFP expression in developing testes from *Uchl1-eGfp* mice during the first round, or wave, of spermatogenesis (P0, P1, P8, P12, P14, P17, P30). All germ cells were EGFP^+^/UCHL1^+^ in P0, P1, P8, P12 and P14 testes (Fig. 4A-C′,F-H′, Fig. S5A-D, Fig. S6A-H). In P17 testes, although all UCHL1^+^ cells were EGFP^+^, EGFP also became detectable in cytoplasmic projections of Sertoli cells (Fig. 4D,D′, Fig. S6D). This was confirmed by co-immunostaining with the Sertoli cell marker GATA4; 12.5% of GATA4^+^ Sertoli cells were also EGFP^+^, with varying epifluorescence intensity (Fig. 4I,I′, Fig. S6I). The percentage of EGFP^+^ Sertoli cells increased over time, such that by P30 nearly all tubules had dim EGFP immunostaining in Sertoli cytoplasm, and 100% of Sertoli cell nuclei were EGFP^+^ (Fig. 4E,E′J,J′, Fig. S6J). Taken together, we conclude that EGFP expression in *Uchl1-eGfp* testes was restricted to germ cells until P14, became detectable in a subset of Sertoli cells starting at P17 and was in all Sertoli cells by P30.

The appearance of few EGFP^+^ Sertoli cells as early as P17 in *Uchl1-eGfp* testes is roughly coincident with their terminal differentiation (França et al., 2016; Griswold, 1998, 2018). We examined this potential link to terminal differentiation by immunostaining P14, P17 and P30 testes for marker of proliferation Ki-67 (MKI67). There was no apparent correlation between the expression of MKI67 and EGFP in Sertoli cells – all were MKI67^−^ (suggesting terminal differentiation) on P14, yet only a subset (12.5%) was EGFP^+^ on P17 (Fig. S7A-C).

EGFP was readily detectable in basally located spermatogonia in *Uchl1-eGfp* testes, but its levels decreased in centrally located spermatocytes (Figs 3, 4). To confirm the identities of EGFP^bright^ and EGFP^dim^ TRA98^+^ germ cells, we immunostained P8 testes for cadherin 1 (CDH1), an established protein marker of undifferentiated spermatogonia (Tokuda et al., 2007), and the differentiation marker KIT. P8 testes also contain the first STRA8^+^ preleptotene spermatocytes entering meiosis (Bellve et al., 1977; Oakberg, 1956b; Roosen-Runge, 1952; Sertoli, 1872), which are located more centrally in testis cords. At P8, all CDH1^+^ and KIT^+^ spermatogonia were EGFP^+^ (Fig. S8A-B″). Interestingly, both CDH1^+^ and KIT^+^ spermatogonia had bright EGFP epifluorescence (EGFP^bright^), whereas CDH1^−^/KIT^−^/STRA8^+^ preleptotene spermatocytes had dim EGFP epifluorescence (EGFP^dim^) (Fig. S8A-B″).

### Isolation of millions of EGFP^+^ spermatogonia at each stage of their development

**Fig. 5.**
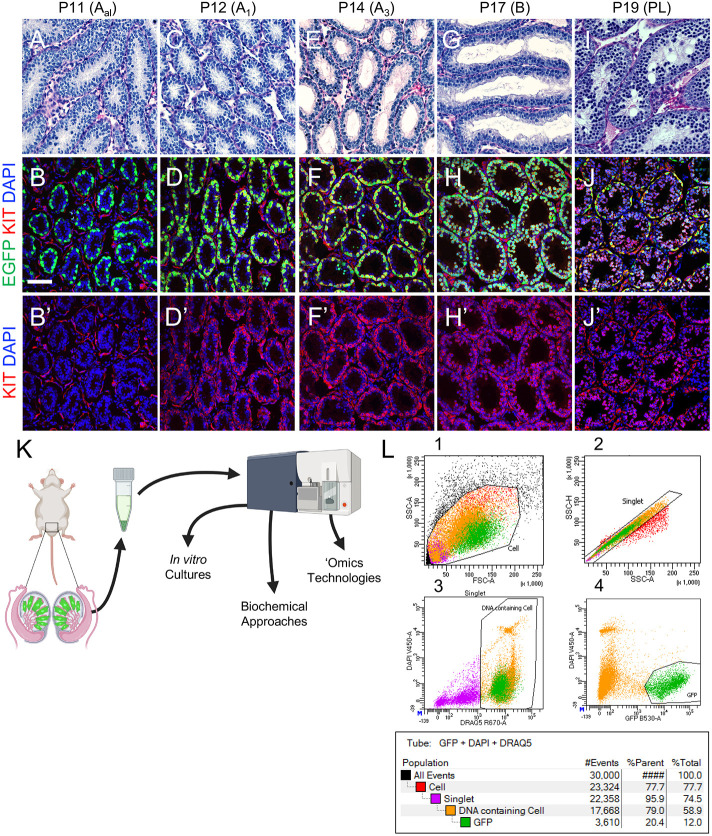
**Fluorescence-activated cell sorting (FACS)-based isolation of millions of EGFP^bright^ spermatogonia and EGFP^dim^ preleptotene spermatocytes.** (A-J′) Testes were harvested from mice with synchronized spermatogenesis (as in Fig. 1A), and each column represents a different age (indicated above). The top row includes periodic acid–Schiff-stained testes, and the middle and bottom rows include testes with EGFP (green) and immunostained for KIT (red), with nuclei labeled in blue. (K,L) Testis single-cell suspensions were generated from P17 *Uchl1-eGfp* mice with synchronized spermatogenesis and used for FACS to isolate EGFP^+^ cells. Graph L1 shows gating for all cells. Graph L2 shows gating for singlets. Graph L3 shows gating for DRAQ5 (intact cells) on the *x*-axis and DAPI (viability) on the *y*-axis. Graph L4 shows gating for EGFP. Scale bar: 50 µm. Cell sorts for each developmental age represented above were performed five times (*n*=5) as separate biological replicates using separate animals. Four (*n*=4) *Uchl1-eGfp*^+^ males were used for each sort.

We again synchronized spermatogenesis in *Uchl1-eGfp* mice to isolate highly enriched viable populations of both spermatogonia with histologically confirmed identity at each stage of their development as well as preleptotene spermatocytes (Fig. 1A). Testes from WIN 18,446-treated mice contained only undifferentiated spermatogonia that were ZBTB16^+^/KIT^−^/SYCP3^−^ (Fig. 5A-B′, Fig. S9A-C), the majority of which became KIT^+^ type A_1_ differentiating spermatogonia on P12, 24 h after RA injection (Fig. 5C-D′, Fig. S9D-F). Over the next several days, these KIT^+^ spermatogonia proliferated, gradually lost ZBTB16 expression, as shown previously (Niedenberger et al., 2015), and progressed through the stages of differentiation (Fig. 5E-H′, Fig. S9G-L) before initiating meiosis on P19 as preleptotene spermatocytes (Fig. 5I-J′, Fig. S9M-O), a timeline matching differentiation during adult steady-state spermatogenesis. Quantitation for each marker at each age is shown in Fig. S9P.

This fluorescent model offers two key advantages over those existing in the literature (Gewiss et al., 2021; Romer et al., 2018): (1) *Uchl1-eGfp* mice were outbred onto a CD-1 background, and thus have large litters, with three to five males per litter (50% of which are transgenic), allowing for isolation of millions of EGFP^+^ germ cells from a single litter; and (2) preleptotene spermatocytes were EGFP^dim^ (Fig. S9O), enabling their isolation in large numbers for the first time. It is crucial to note that Sertoli cells do not express EGFP until ∼P20 in the synchronized *Uchl1-eGfp* testes, compared with onset of EGFP expression at P17 in unsynchronized *Uchl1-eGfp* testes. To isolate highly enriched populations of differentiating types A_1_, A_3_ and B spermatogonia, and preleptotene spermatocytes, testis single-cell suspensions were generated from mice at specific ages based on the established developmental timeline (Fig. 1A) and used for FACS (Fig. 5K,L). EGFP^+^ sorted germ cells from mice aged P12, P14, P17 and P19 were 91-94% pure (Table 1), as assessed by purity analysis via flow cytometry (Fig. S10A) as well as by staining an aliquot of sorted cells with KIT, an established protein marker of differentiating spermatogonia (Fig. S10B,B′, 94% of EGFP^+^ cells were KIT^+^). Following each FACS experiment, the viability of sorted germ cell populations was assessed in two ways: (1) small aliquots were used for viability assessment via flow cytometry (Fig. 5L); and (2) germ cells were pipetted into a 96-well (=100,000 cells/well) and stained with tetramethylrhodamine (TMRM), a cell-permeant fluorescent dye sequestered only within active mitochondria (Fig. S10C,C′).

**Table 1.**
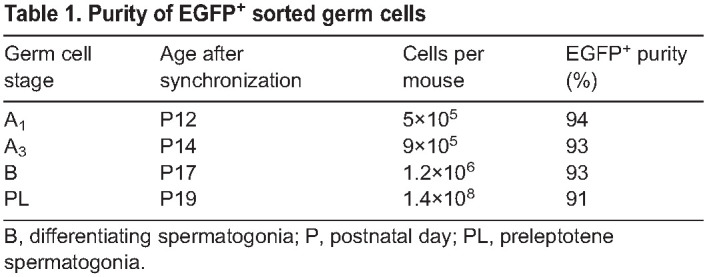
Purity of EGFP^+^ sorted germ cells

### Proteomic analysis identifies novel markers of the mitosis-to-meiosis transition

**Fig. 6.**
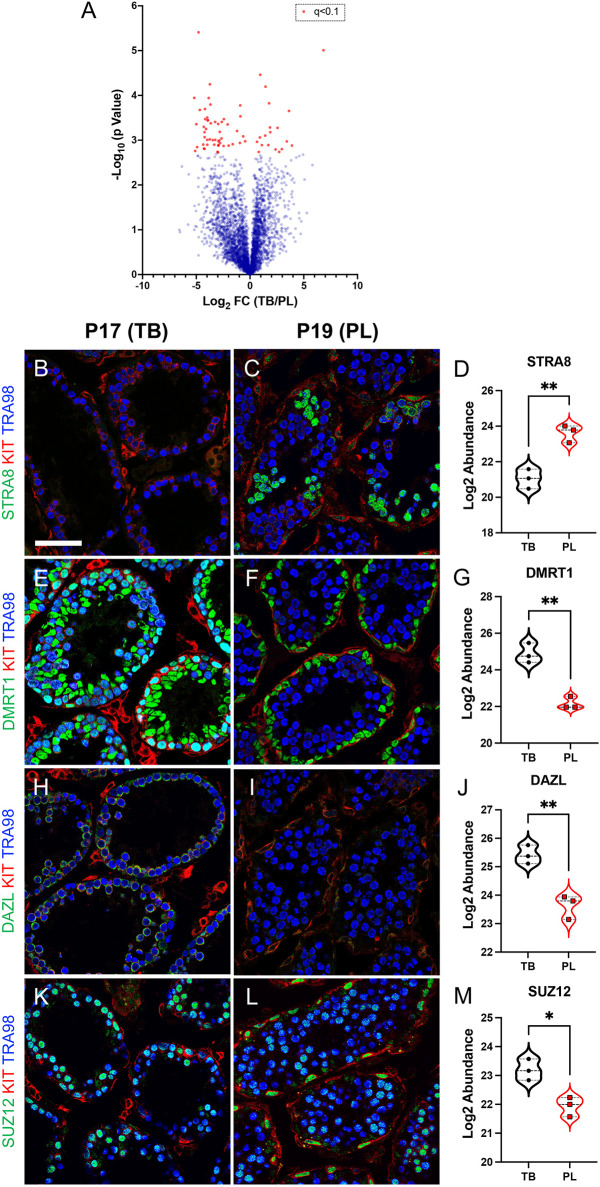
**Mass spectrometry-based shotgun proteomics identified bona fide protein markers of male germ cell fate.** (A) Volcano plot depicting changes in proteome abundance between mitotic type B differentiated spermatogonia (TB) and meiotic preleptotene spermatocytes (PL). Red circles represent differentially expressed proteins using an adjusted *P*-value cutoff of *q*<0.1. (B-M) Immunostaining and associated proteomics values for specific proteins (STRA8, DMRT1, DAZL and SUZ12); the colors representing each detected protein are shown to the left of each image. The left column of images represents P17 testes (containing TB spermatogonia), and the right column of images represents P19 testes (containing PL spermatocytes). Scale bar: 50 µm. The experiment was done using three (*n*=3) biological replicates for each developmental age. Separate FACS-based isolations from four (*n*=4) animals were performed to obtain cell populations for each biological replicate used for proteomics.

Finally, we performed the first ever, to the best of our knowledge, label-free shotgun proteomics screen on meiotic preleptotene spermatocytes and their immediate predecessors, mitotic type B differentiated spermatogonia undergoing steady-state spermatogenesis. This approach was chosen to perform a technique requiring millions of cells at these stages to study differentiation and meiotic initiation, as well as to identify novel germ cell protein markers during meiotic initiation. Across all samples, 3550 individual proteins were identified and quantified (see Table S1 for all data). Using an adjusted *P* value of *q*<0.1, 70 proteins were differentially expressed between type B differentiated spermatogonia to meiotic preleptotene spermatocytes (Fig. 6A). From the list of differentially expressed proteins, we selected several candidates for confirmation by immunostaining synchronized testes from P17 and P19 mice. As expected from previous reports (Lei et al., 2007; Matson et al., 2010) and our proteomics results, STRA8 protein levels increased (Fig. 6B-D) and DMRT1 levels decreased (Fig. 6E-G) in germ cells from P17 (type B differentiating spermatogonia) to P19 (preleptotene spermatocytes). In addition, we confirmed proteomics results for DAZL and SUZ12 (Fig. 6H-M).

## DISCUSSION

Here, we report two physiologically relevant models intended to enable a broad userbase of scientists to study the enigmatic developmental processes of mammalian spermatogonial differentiation and meiotic initiation that are crucial for spermatogenesis and male fertility. First, we developed a facile method to model spermatogonial differentiation and meiotic initiation *in vitro* that can be readily adapted by numerous laboratories. Second, we identified a transgenic mouse model with EGFP^+^ spermatogonia that can be used in a straightforward synchronization protocol for the FACS-based isolation of numerous prospermatogonia, spermatogonia and preleptotene spermatocytes. Using differentiating spermatogonia and preleptotene spermatocytes at precise histologically defined stages of development, we performed mass spectrometry (MS)-based shotgun proteomics to define *in vivo* protein-level changes in gene expression. With these gene expression signatures as a guide, we validated a panel of protein markers that can be detected by immunostaining to assess the faithful completion of spermatogonial differentiation and meiotic initiation *in vitro*.

### Applicability of this synchronization model to study adult steady-state spermatogenesis

Because spermatogonia represent a heterogeneous and rather small percentage of the germ cell population in the adult testis, they are incredibly difficult to study. Because of this, researchers in the past have studied mouse spermatogonia development in the first week of life during what has been called the first round, or wave, of spermatogenesis (Geyer, 2017). It is during this time that a heterogeneous population of stem, progenitor and differentiating spermatogonia emerge from quiescent precursor prospermatogonia at ∼P3 (Agrimson et al., 2016; Kluin and de Rooij, 1981; Kluin et al., 1984; Velte et al., 2019). One longstanding concern in the field regarding use of the first wave as a valid model to study adult steady-state spermatogonia is that the differentiation program is shortened. In adult steady-state spermatogenesis, it takes ∼8 days from RA-initiated differentiation to formation of preleptotene spermatocytes (Oakberg, 1956b; Roosen-Runge, 1952). In contrast, the differentiation program in first-wave spermatogonia lasts only ∼6 days (Kluin et al., 1982). This truncated timeline was also presumed in a recent study using hybrid C57Bl/6x129 synchronized mice treated with WIN 18,446 to P8 (here we treated to P10, owing to our observation of small populations of STRA8^−^ preleptotene spermatocytes in WIN 18,446-treated mice, shown in Fig. S2D-F). However, in that study, cultures were not extended to formation of meiotic spermatocytes, so it is difficult to conclude the absolute length of differentiation (Agrimson et al., 2016). Here, preleptotene spermatocytes formed ∼8 days after RA administration, mimicking the timeline of adult steady-state spermatogenesis.

A subset of Sertoli cells became EGFP^+^ at P17, roughly coincident with their terminal differentiation. Because there was no apparent correlation between terminal differentiation and EGFP status, it is possible that Sertoli cells were EGFP^+^ due to phagocytosis of EGFP^+^ germ cells, which is consistent with previous reports of the onset of endogenous UCHL1 protein expression in Sertoli cells (Kon et al., 1999). This feature may allow simultaneous FACS-based isolation and co-culture of EGFP^+^ spermatogonia and Sertoli cells.

### Comparing the *in vitro* differentiation and meiotic initiation results in primary cultures with recent *in vitro* studies with long-term cultures

Recently, it was reported that the RA-stimulated process of spermatogonial differentiation either could not be recapitulated *in vitro* (Sinha et al., 2021) or required ‘nutrient restriction’ (Zhang et al., 2021). A feature common to both studies was that differentiation was initiated by adding RA to relatively pure cultures of spermatogonia maintained for weeks to months in media containing high levels of growth factors GDNF and FGF2. Importantly, these studies used culture-adapted primary spermatogonia that were maintained in the presence of two recombinant growth factors, but the absence of normal testicular somatic cells, such as Sertoli and peritubular myoid cells, which may play key roles in advancing spermatogonial differentiation independent of the RA signal. Among the many possible factors, KITL [also termed stem cell factor (SCF)] is produced by Sertoli cells and essential for survival and proliferation of differentiating spermatogonia (Ohta et al., 2000; Packer et al., 1995; Yan et al., 2000). Indeed, in this report, we found that freshly isolated spermatogonia could initiate and complete differentiation and enter meiosis *in vitro*, in the presence of GDNF and FGF and in nutrient-rich media in response to exogenous RA. Overall, long-term cultures of spermatogonia in an undifferentiated state appear to have significantly reduced capacity to differentiate and enter meiosis, and thus may not represent an optimal model for these essential developmental programs.

### Wide applicability of modeling spermatogonia and meiotic initiation *in vitro*

The simplified culture system model presented here utilizes single-cell suspensions from whole testes of mice with synchronized spermatogenesis at any point following spermatogonial differentiation. Because spermatogonia closely associate with Sertoli cells in culture (as *in vivo*), this obviates the need for embryonic cell feeder layers. In this system, spermatogonia completed the process of differentiation and both initiated and progressed through the first steps of meiosis. The presented proteome profiles of premeiotic type B differentiating spermatogonia and meiotic preleptotene spermatocytes are a resource for scientists to examine new gene products and signaling pathways during differentiation that uniquely prepare spermatogonia for meiosis. In particular, the use of *Uchl1-eGfp* mice for facile isolation of millions of spermatogonia and preleptotene spermatocytes at each stage of their development opens the door for a multitude of unbiased genome-wide applications.

An additional application of these approaches is the identification of male contraceptive therapeutic drug targets. Currently, there are no approved safe, effective and reversible oral male contraceptives, constituting a significant unmet need for population control worldwide. Historically, steroidal male contraceptives have proven difficult to commercialize, and non-steroidal male contraceptive drugs have met with poor success. This failure is in part due to efforts focused almost solely on testis-specific proteins expressed in spermatocytes and spermatids residing adluminal to the blood–testis barrier (BTB). Because the BTB inhibits drug delivery (Cheng and Mruk, 2012), efforts should instead be focused on identifying druggable targets (and their candidate inhibitor compounds) expressed outside the BTB, such as in differentiating spermatogonia and preleptotene spermatocytes. Both cell populations are distinct from the SSC pool; therefore, differentiating spermatogonia and preleptotene spermatocytes represent ideal cell types that express potential targets for development of safe, non-surgical, non-hormonal and reversible male contraceptive compounds.

## MATERIALS AND METHODS

### Animal care

All procedures using animals adhered to guidelines outlined in the National Research Council Guide for the Care and Use of Laboratory Animals and were approved by the Animal Care and Use Committee at East Carolina University (approval A3469-01). *Uchl1-eGfp* mice were created by the Ozdinler laboratory (Yasvoina et al., 2013) and obtained from The Jackson Laboratory (stock no. 022476) on a C57Bl/6 genetic background and outcrossed with CD-1 mice (Charles River Laboratories). These and CD-1 mice were used for this study, and the day of birth was designated as P0. Mice were humanely euthanized by decapitation prior to P7, and by CO_2_ asphyxiation followed by cervical dislocation after P7.

### Synchronizing spermatogenesis in the developing testis

Spermatogenesis was synchronized similarly to previous reports (Hogarth et al., 2013; Romer et al., 2018). Briefly, the RA synthesis inhibitor *bis*-(dichloroacetyl)-diamine/WIN18,446 (14018, Cayman Chemical) was resuspended in vehicle [dimethyl sulfoxide (DMSO), final concentration 100 µg/g of body weight] and fed to pups daily from P1 to P10 using a 24-gauge blunt metal feeding needle. This WIN18,446 treatment was discontinued on P11, and mice received one subcutaneous injection of 10 µl exogenous RA (10 µg/µl) in DMSO to initiate spermatogonial differentiation. Males with synchronized spermatogenesis were euthanized at P12 for isolation of type A_1_ spermatogonia, at P14 for isolation of type A_3_ spermatogonia, at P17 for isolation of In/type B spermatogonia and at P19 for isolation of preleptotene spermatocytes.

### Testis explant culture system

Testes were removed and placed in Hanks’ buffered saline solution (HBSS) for detunication. Testes were then minced into pieces small enough to fit within a singular drop of medium. Pieces were placed on the underside of the well plate lid, and 40 µl charcoal-stripped solution medium (89% a-MEM with Glutamax, 10% CSS, 1% penicillin-streptomycin) was added to each sample. Well plate lids were carefully reverted and placed over 24-well plates with wells filled with 200 µl sterile PBS. Culture medium was replenished every other day until the time of tissue collection.

### Single-cell suspensions

Single-cell testis suspensions were generated as before (Chappell et al., 2013). Briefly, testes were removed and placed in HBSS. For each experiment, testes from *n*≥3 adult males or three to six pups from a given litter were detunicated and transferred to a solution containing 4.5 ml of 0.25% trypsin (Gibco) and 0.5 ml DNase1 (7 mg/ml, Sigma-Aldrich) and incubated in a 37°C water bath for 3 min. Then, an additional 1 ml DNase1 was added and incubation continued for a further 3 min. An additional 1 ml DNase1 was added, and the mixture was triturated to further break up the testes, followed by a 1 min incubation in a 37°C water bath. One milliliter of fetal bovine serum (FBS; ATCC) was added to deactivate the trypsin, and the mixture was triturated to break apart any remaining intact testis clumps. The cell mixture was filtered through a 40 μm sieve and centrifuged at 600 ***g*** for 7 min. The resulting cell pellet was suspended in sorting medium containing 3% FBS+10 mM EDTA+10 mM HEPES in 1× PBS and used for downstream flow cytometry experiments.

### *In vitro* spermatogenesis culture

Single-cell testis suspensions were suspended in 10% charcoal-stripped FBS (A3382101, Thermo Fisher)+1% penicillin-streptomycin (Thermo Fisher, #15070063)+DMEM/F12 (10565018, Thermo Fisher)+1 µM RA (R2625, Sigma-Aldrich) and cultured on glass bottom 96-well plates (P96-1.5H-N, Cellvis) with 175,000 cells per well. Media included combinations of the following: 1 µM RA (R2625, Sigma-Aldrich), 15 ng/ml rat GDNF (450-51, Peprotech) and 10 ng/ml human FGF2 (100-18B, Peprotech). Suspensions were cultured at 34°C with 5% CO_2_ and media were changed daily. After culture, cells were fixed with 4% paraformaldehyde in 1× PBS.

### FACS

EGFP^+^ germ cells were sorted on a Becton Dickinson AriaFusion cell sorter. A 100 mW 488 nm laser was used for excitation of the EGFP signal, and a 530/30 bandpass filter for detection of the emitted fluorescence. Dead cells were removed using forward and side scatter gating as well as fluorescence gating on cells excluding 4′,6-diamidino-2-phenylindole (DAPI; D1306, Thermo Fisher) fluorescence. Single DNA-containing cells were identified using DRAQ5 fluorescent probe (564902, BD Pharmingen). Doublets were gated out using forward scatter (FSC)-height versus FSC-area plots. An 85 µm nozzle was used for sorting, and cell flow rate was controlled between 5000-9000 events/s. For isolation of EGFP^bright^ populations, the median fluorescence intensity (MFI) values were in a ∼26,000-28,000 range. For isolation of EGFP^dim^ population, the MFI values were ∼5250, approximately 80% lower than MFIs of EGFP^bright^. MFIs were taken from a single log-normal distribution for each sort. It is important to note that MFI values were generated using the equipment and software above; users should use these values as guides for proper optimization of their instruments. Cells were sorted into sorting buffer (15% FBS+10 mM EDTA+10 mM HEPES in 1× PBS) in 5 ml polypropylene tubes (352063, Corning LS) precoated with 10% BSA. Cell purity was assessed by reanalyzing a small aliquot of the sorted cells via flow cytometry as well as by staining a small aliquot of sorted cells for specific protein cell fate markers.

### Histology and indirect immunofluorescence (IIF)

For histological analyses, whole testes were immersion fixed in Bouin's solution for 24 h at 4°C, washed overnight in 1× PBS, dehydrated through an ethanol series, processed using standard methods and then embedded in paraffin. Sections (5 µm) were cut and stained with the periodic acid–Schiff method using standard methods, and images were taken on an Axio Observer A1 microscope (Carl Zeiss Microscopy, LLC) outfitted with Axiocam 503 color camera and Zen software (Carl Zeiss Microscopy, LLC).

For IIF, testes were immersion fixed for 24 h at 4°C in fresh 4% paraformaldehyde, washed overnight in 1× PBS, and incubated in 30% sucrose at 4°C for 24 h. Testes were frozen in O.C.T. compound, and 5 μm cryosections were cut for immunostaining. Primary antibodies used were as follows: anti-TRA98 (1:1000, ab82527, Abcam, rat monoclonal), anti-GFRA1 (1:800, AF560, R&D Systems, goat polyclonal), anti-KIT (1:1000, AF1356, R&D Systems, goat polyclonal), anti-ZBTB16 (1:2000, ab189849, Abcam, rabbit polyclonal), anti-UCHL1 (1:1000, 13179S, Cell Signaling Technology, rabbit monoclonal), anti-DAZL (1:200, ab215718, Abcam, rabbit monoclonal), anti-SUZ12 (1:800, 3737S, Cell Signaling Technology, rabbit monoclonal), anti-γH2AX (1:400, ab11174, Abcam, rabbit polyclonal), anti-GATA4 (1:1000, 36966S, Cell Signaling Technology, rabbit monoclonal), anti-STRA8 (1:3000, ab49602, Abcam, rabbit polyclonal), anti-CDH1 (1:1000, 3195S, Cell Signaling Technology, rabbit monoclonal) and anti-DMRT1 (1:1000; Lei et al., 2007). Primary antibody was omitted in negative controls. Following stringency washes, sections were incubated in secondary antibodies (1:500, Alexa Fluor donkey anti-rabbit-488, Alexa Fluor donkey anti-goat-488, Alexa Fluor donkey anti-goat-555, Alexa Fluor donkey anti-rat-555, Thermo Fisher). Fluorescently conjugated anti-SYCP3-488 (1:200, ab205846, Abcam), Phalloidin-635 (1:500, A34054, Life Technologies) and Lectin-488 (1:500, L21409, Thermo Fisher) were applied without secondary antibodies. Coverslips were mounted with Vectastain containing DAPI (Vector Laboratories) or 1:1 PBS:glycerol solutions, and images were obtained using a Fluoview FV1000 confocal laser-scanning microscope (Olympus America). Testes from *n*≥3 different mice were analyzed for each experiment, and immunostaining was repeated at least twice, for each marker.

### MS-based shotgun proteomics

From *Uchl1-eGfp* mice with synchronized spermatogenesis, FACS was used to isolate 3-6×10^6^ EGFP^+^ spermatocytes at the mid-point of preleptonema and their immediate predecessors, type B differentiating spermatogonia. Experimental samples (*n*=3, each) were lysed in urea lysis buffer (8 M urea in 40 mM Tris-HCl, 30 mM NaCl, 1 mM CaCl_2_, 1× cOmplete ULTRA mini EDTA-free protease inhibitor tablet; pH 8.0), as described previously (McLaughlin et al., 2020; Smith et al., 2020). The samples were subjected to two freeze-thaw cycles and sonicated in three 5 s bursts (Q Sonica CL-188; amplitude of 30). Samples were centrifuged at 10,000 ***g*** for 10 min at 4°C. Protein concentration was determined by BCA. Equal amounts of protein were reduced with 5 mM DTT at 37°C for 30 min, and then alkylated with 15 mM iodoacetamide for 30 min in the dark. Unreacted iodoacetamide was quenched with DTT (15 mm). Reduction and alkylation reaction were carried out at room temperature. Initial digestion was performed with Lys C (1:100 w/w) for 4 h at 32°C. Following dilution to 1.5 M urea with 40 mM Tris-HCl (pH 8.0), 30 mM NaCl, 1 mM CaCl_2_, samples were digested overnight with sequencing grade trypsin (50:1 w/w) at 32°C. Samples were acidified to 0.5% trifluoroacetic acid and then centrifuged at 4000 ***g*** for 10 min at 4°C. Supernatant containing soluble peptides was desalted, as described previously (McLaughlin et al., 2020; Smith et al., 2020), and then eluate was frozen and subjected to Speedvac vacuum concentration.

### Nanoscale liquid chromatography – tandem mass spectrometry (nanoLC-MS/MS) for label-free proteomics

Final peptides were resuspended in 0.1% formic acid, quantified (23275, Thermo Fisher), and then diluted to a final concentration of 0.25 µg/µl. Samples were subjected to nanoLC-MS/MS analysis using an UltiMate 3000 RSLCnano system (Thermo Fisher) coupled to a Q Exactive Plus Hybrid Quadrupole-Orbitrap mass spectrometer (Thermo Fisher) via a nanoelectrospray ionization source. For each injection, 4 µl (1 µg) of sample was first trapped on an Acclaim PepMap 100 20 mm×0.075 mm trapping column (164535, Thermo Fisher; 5 μl/min at 98/2 v/v water/acetonitrile with 0.1% formic acid). Analytical separation was performed over a 95 min gradient (flow rate of 250 nl/min) of 4-25% acetonitrile using a 2 µm EASY-Spray PepMap RSLC C18 75 µm×250 mm column (ES802A, Thermo Fisher) with a column temperature of 45°C. MS1 was performed at 70,000 resolution, with an automatic gain control (AGC) target of 3×10^6^ ions and a maximum injection time (IT) of 100 ms. MS2 spectra were collected by data-dependent acquisition of the top 15 most abundant precursor ions with a charge greater than 1 per MS1 scan, with dynamic exclusion enabled for 20 s. The precursor ion isolation window was 1.5 m/z, and normalized collision energy was 27. MS2 scans were performed at 17,500 resolution, maximum IT of 50 ms and AGC target of 1×10^5^ ions.

### Data analysis for label-free proteomics

As described previously (McLaughlin et al., 2020; Smith et al., 2020), Proteome Discoverer 2.2 (PDv2.2) was used for raw data analysis, with default search parameters including oxidation (15.995 Da on M) as a variable modification and carbamidomethyl (57.021 Da on C) as a fixed modification. Data were searched against the UniProt *Mus musculus* reference proteome (Proteome ID: UP000000589). Peptide-spectrum matches were filtered to a 1% false discovery rate (FDR) and grouped to unique peptides while maintaining a 1% FDR at the peptide level. Peptides were grouped to proteins using the rules of strict parsimony, and proteins were filtered to 1% FDR. Peptide quantification was done using the MS1 precursor intensity. Imputation was performed via low abundance resampling. For all statistical comparisons, multiple hypothesis correction was performed using the Benjamini–Hochberg FDR (Lesack and Naugler, 2011). Protein abundance was normalized to all identified proteins.

### Statistics

Statistical differences between experimental groups were determined using one-way ANOVA, unpaired, one-tailed Student's *t*-test and Tukey's test, with significance levels set at *P*<0.05. Error bars show at least one s.d. Microsoft Excel, GraphPad Prism and QuPath software were used for statistical analyses and generation of graphs in figures.

## Supplementary Material

10.1242/develop.200713_sup1 Supplementary information
