# Supplementary material for: Modeling mammalian spermatogonial differentiation and meiotic initiation in vitro
Source: Development. 2022 Nov 16;149(22):dev200713. doi: 10.1242/dev.200713 (PMC9845750; doi:10.1242/dev.200713)
Supplement: Supplementary information [file develop-149-200713-s1.pdf]

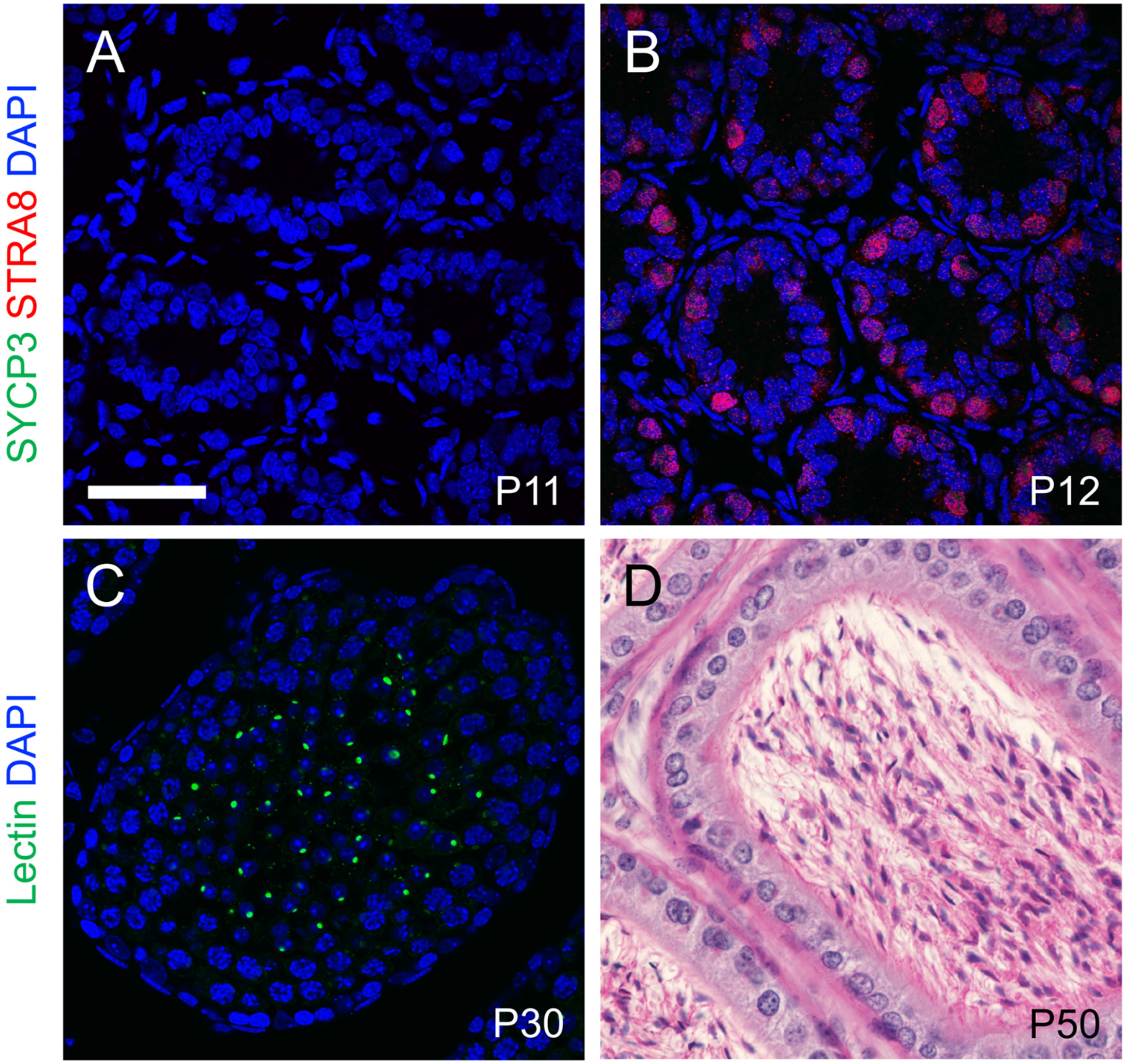

Fig. S1. Validating synchronization of steady-state spermatogenesis *in vivo*. (**A-B**) Immunostaining was done to detect SYCP3 (green) and STRA8 (red) in spermatogonia from testes with synchronized spermatogenesis (Fig. 1A) at P11 before (**A**) and at P12 after (**B**) RA injection. (**C**) Lectin (green) was used to mark the acrosomes of round spermatids in P30 testes. In **A-C**, DAPI marks nuclei (blue). (**D**) Cauda epididymis from P50 mice stained with PAS, with lumina containing sperm. Scale bar = 50 µm. Each representative staining was repeated three times (n=3).

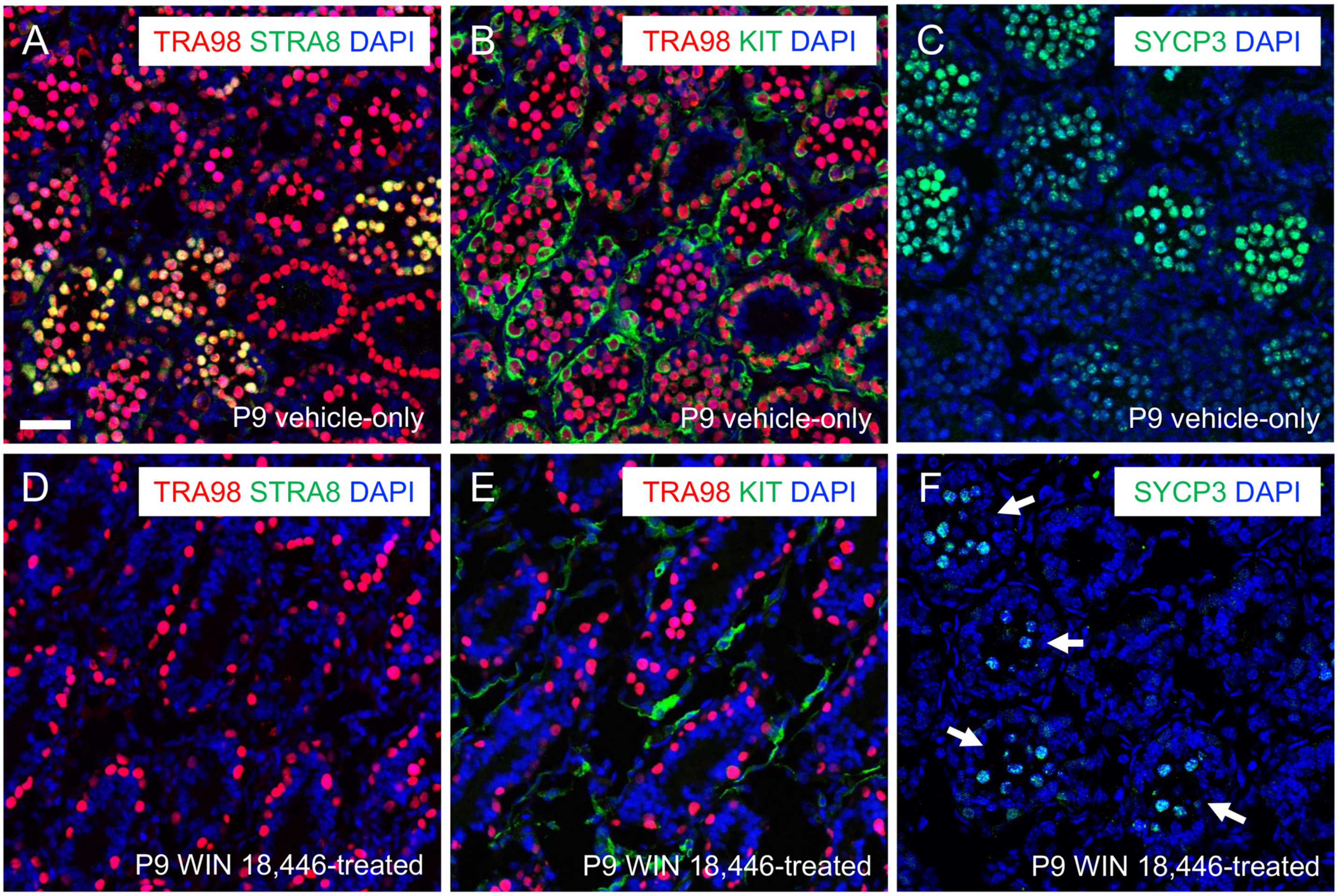

Fig. S2. A small but significant population of male germ cells initiate meiosis in WIN 18,446-treated testes lacking RA during the first wave of spermatogenesis. (**A-F**) Immunostaining was done to colocalize germ cell protein fate markers in P9 WIN 18,446-treated testes (see Fig. 1A for timeline). Top row includes testis images from vehicle alone-treated littermate control mice, while bottom row includes testis images from WIN 18,446-treated mice. (**D-E**) All germ cells in testes from WIN 18,446-treated mice were STRA8-/KIT-, indicating RA deficiency. (**F**) White arrows represent clusters of SYCP3^{ribbon} spermatocytes. Scale bar (in **A**) = 50 µm. Each representative immunostaining was repeated three times (n=3).

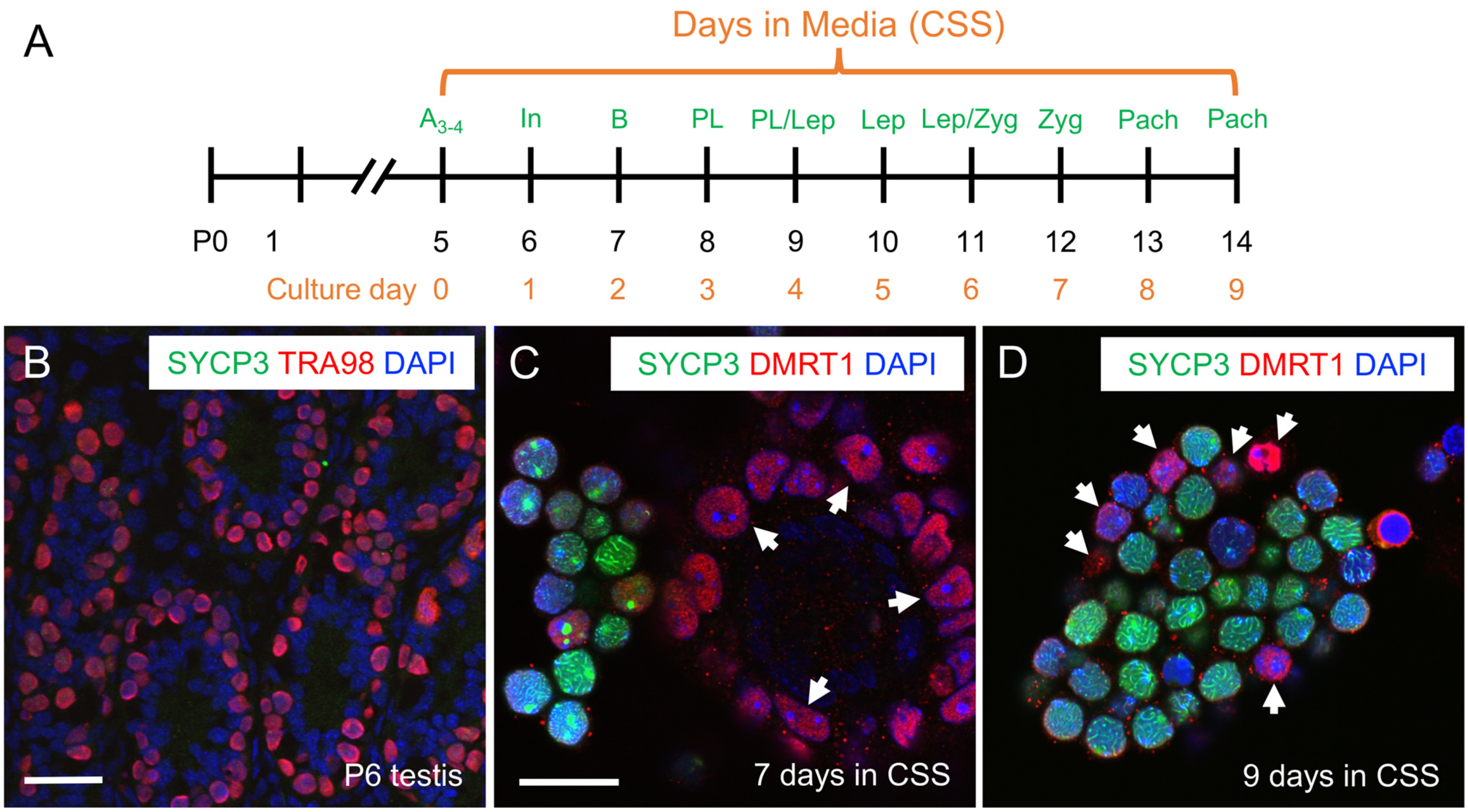

Fig. S3. Neonatal spermatogonia complete differentiation and enter meiosis *in vitro*. (**A**) Timeline for culture experiment. (**B-D**) Immunostaining was done to detect protein markers of germ cell fate, which are indicated with accompanying colors on each image (**B**) Wild-type testes from P6 mice contain only SYCP3- spermatogonia. (**C-D**) At seven (**C**) and nine (**D**) days of culture, DMRT1-/SYCP3^{ribbons} spermatocytes are closely associated with DMRT1+/SYCP3- Sertoli cells, indicated with white arrows. Scale bar (in **A**) = 50 µm. The experiment was repeated three times (n=3) as separate biological replicates. Four technical replicates (n=4) were done for each biological replicate. Each representative immunostaining was repeated three times (n=3).

Development • Supplementary information

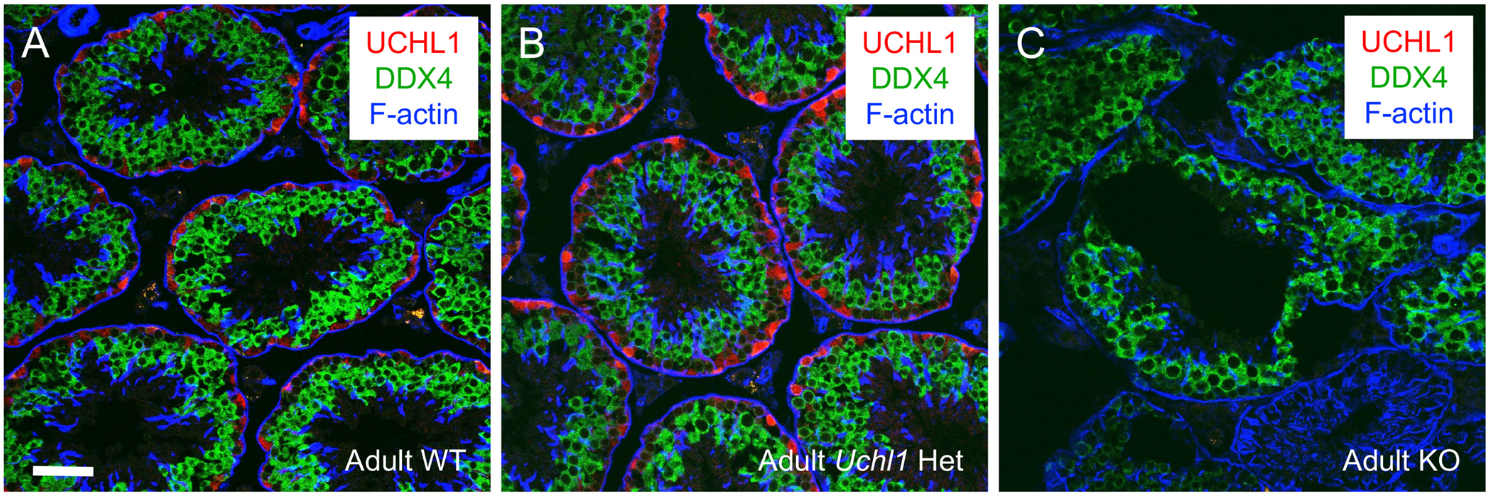

Fig. S4. Validating UCHL1 expression in adult testes. (**A-C**) Immunostaining was done to detect UCHL1 in testes from adult WT (**A**), heterozygous (Het, **B**), and knockout (KO, **C**) testes. DDX4 (in green) marked all germ cells (with highest levels in meiotic spermatocytes), and phalloidin labels the F-actin cytoskeleton (blue). Scale bar = 50 μm. Each representative immunostaining was repeated three times (n=3).

Development • Supplementary information

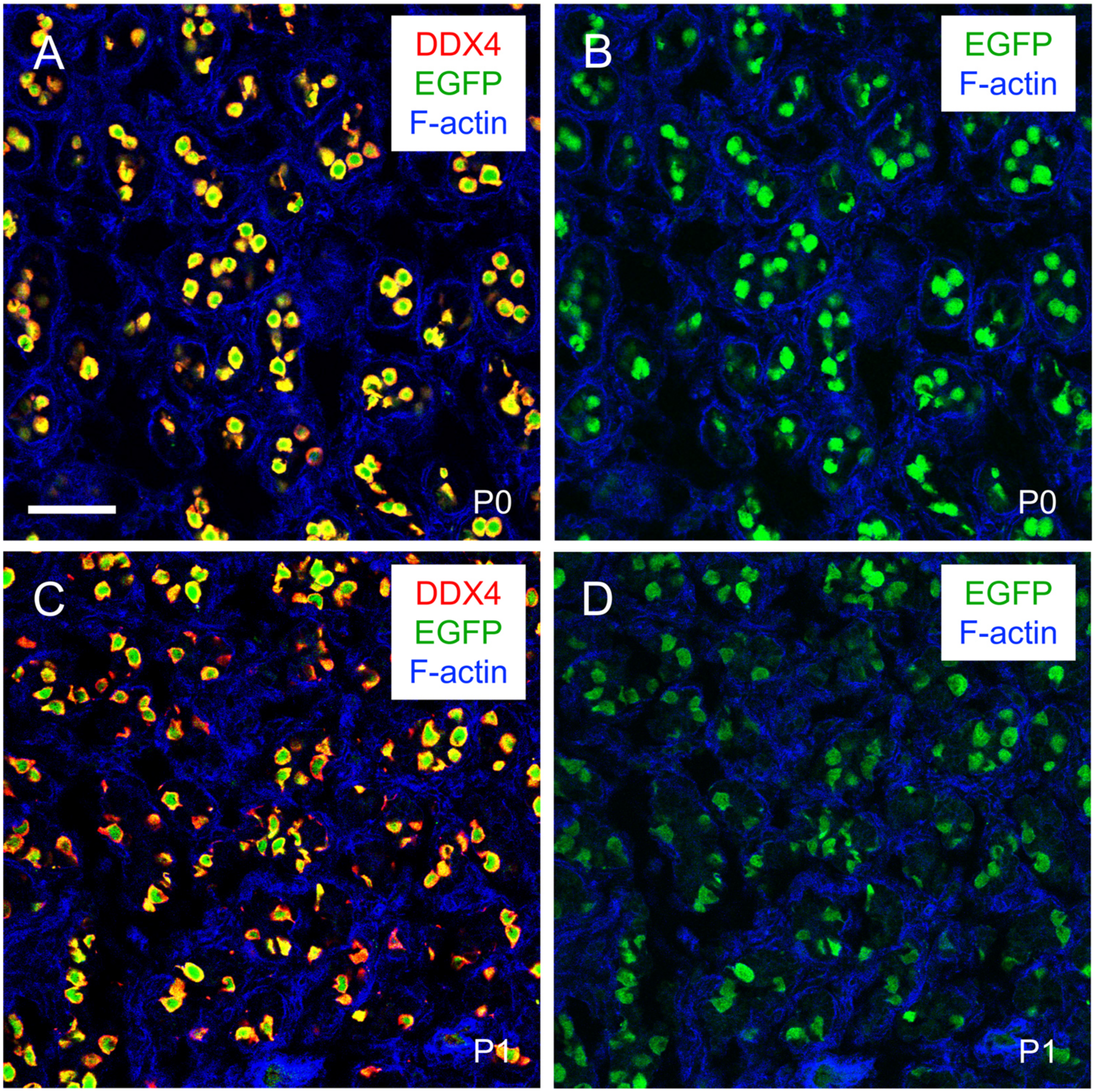

Fig. S5. UCHL1-EGFP is detectable in all prospermatogonia in the developing testis. (**A-D**) Immunostaining was done to colocalize EGFP (green) with DDX4 (red), which is a pan germ cell marker in the developing testis. Phalloidin labeled the F-actin cytoskeleton (blue). Ages are indicated on each image. Scale bars = 25 μm. Each representative immunostaining was repeated three times (n=3).

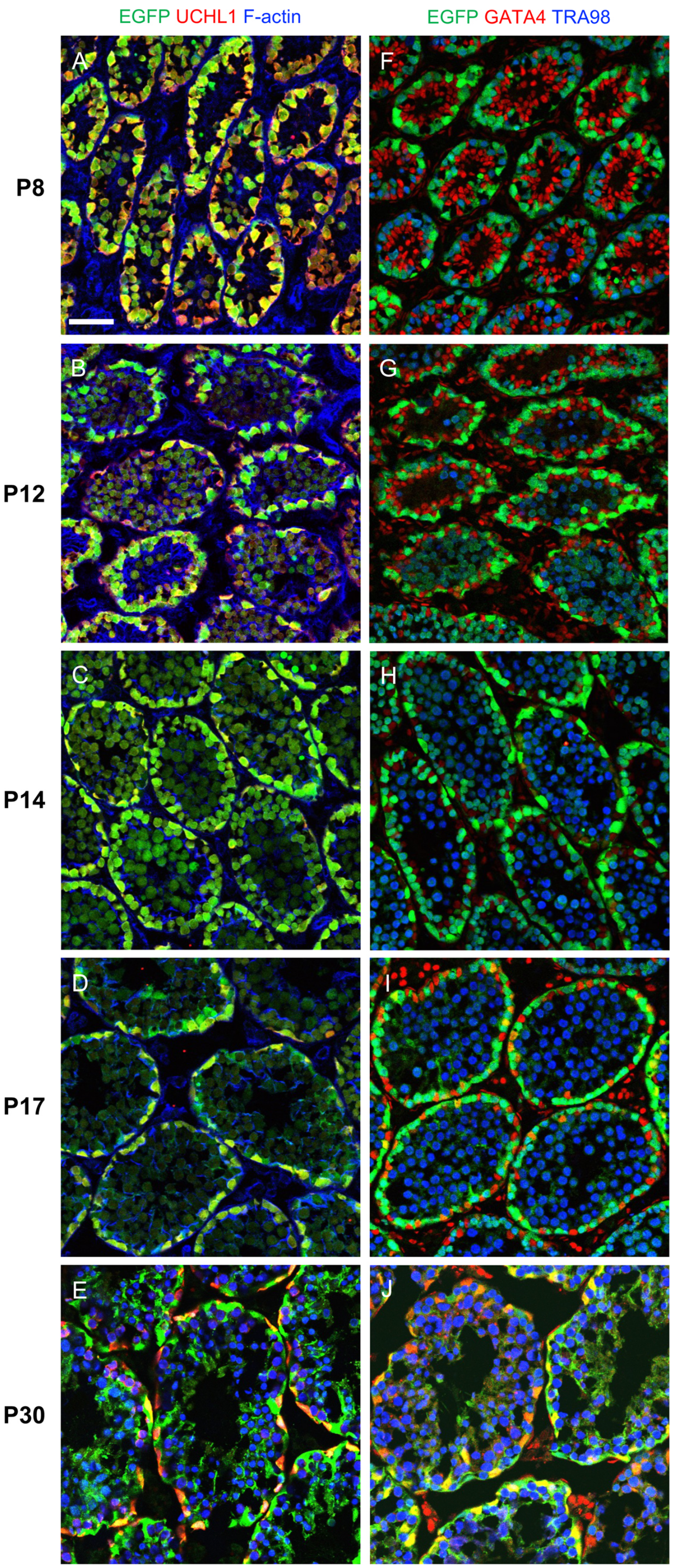

Fig. S6. UCHL1-EGFP expression is restricted to spermatogonia until P14. (**A-J**) Higher magnification images are shown, from the immunostaining results presented in Fig. 4. Immunostaining for protein cell fate markers (indicated, with colors, at the top of each column) for unsynchronized mouse testes during the first wave of spermatogenesis. Mouse ages are shown to the left of each row. Scale bar = 50 µm. Each representative immunostaining was repeated three times (n=3).

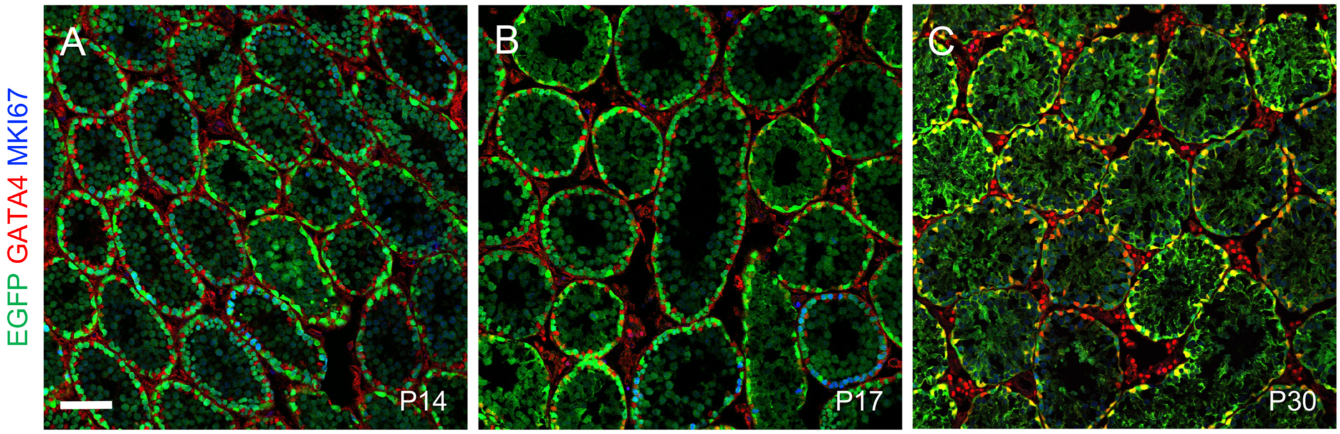

Fig. S7. UCHL1-EGFP expression is not correlated with Sertoli MKI67 status. (**A-C**) Immunostaining was done to colocalize EGFP (green) with GATA4 (red), which is only expressed in Sertoli cells within the seminiferous epithelium, and MKI67 (blue). Ages are indicated on each image. Scale bar (in **A**) = 25 µm. Each representative immunostaining was repeated three times (n=3).

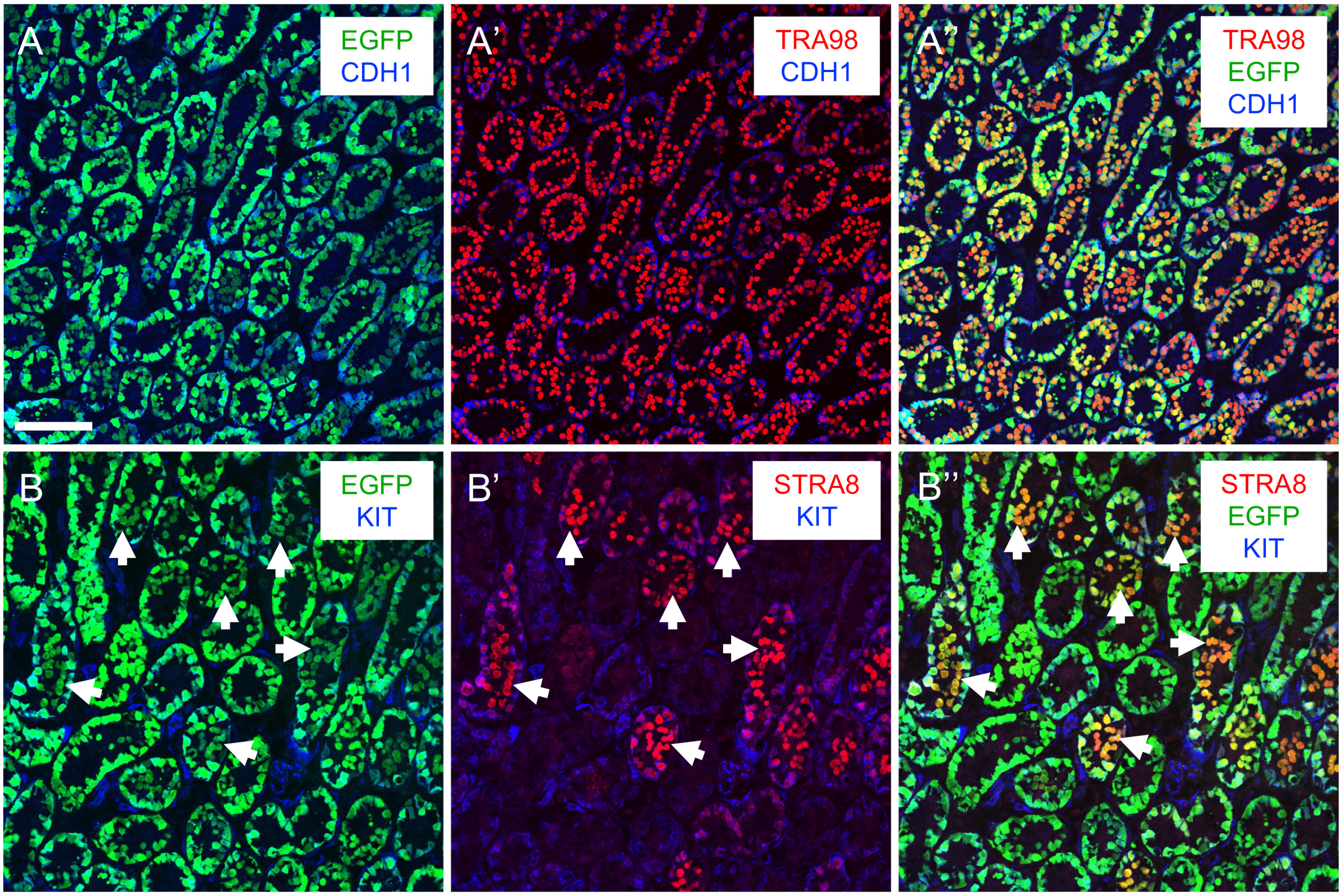

Fig. S8. Spermatogonia are EGFP^{bright} and preleptotene spermatocytes are EGFP^{dim}. (**A-B"**) Immunostaining was done on P8 testes to detect protein fate markers (colors indicated on each image). White arrows (**B-B"**) indicate STRA8+/KIT-/EGFP^{dim} preleptotene spermatocytes. Scale bar = 100 µm. Each representative immunostaining was repeated three times (n=3).

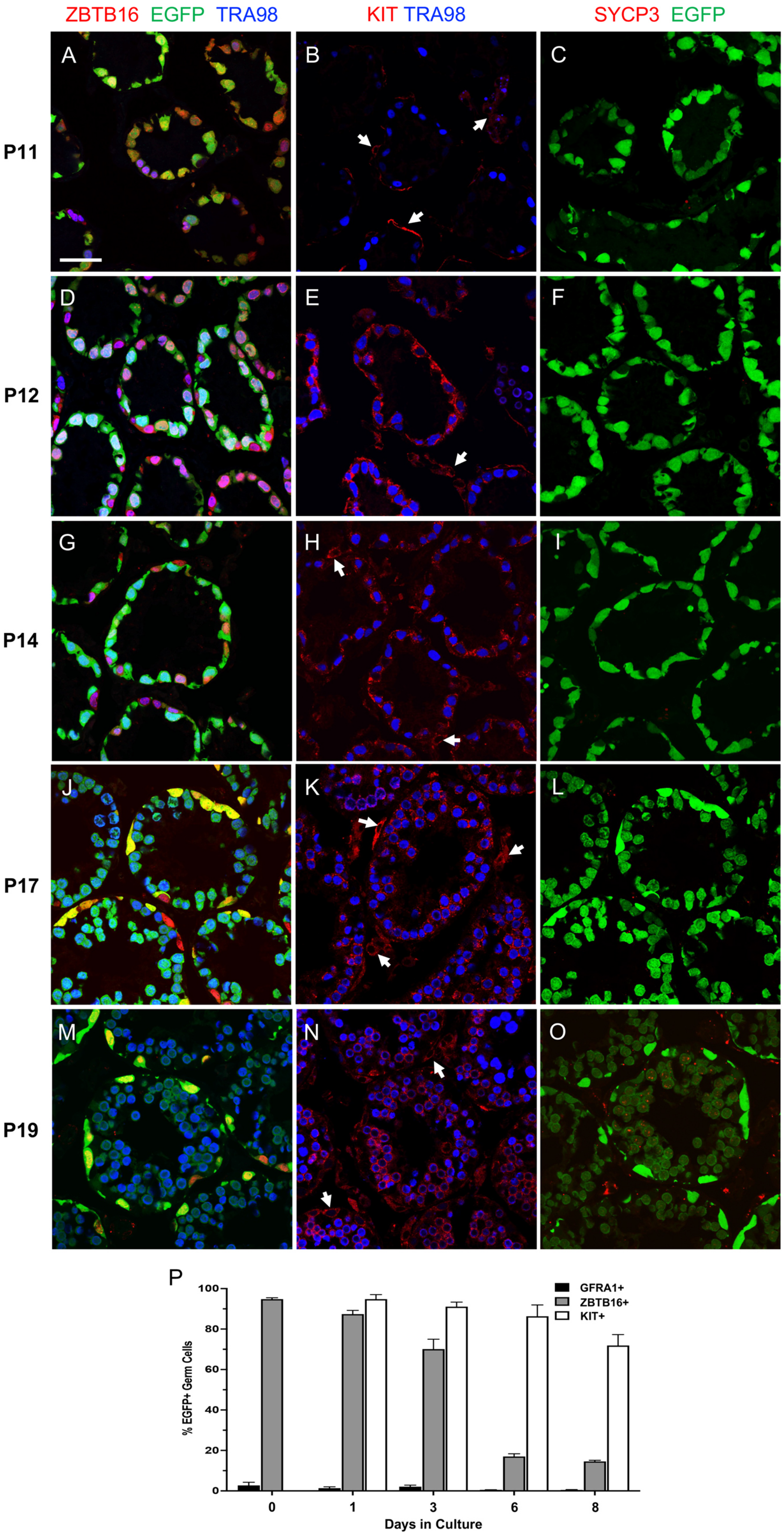

ZBTB16 EGFP TRA98
KIT TRA98
SYCP3 EGFP
P11
P12
P14
P17
P19
A
B
C
D
E
F
G
H
I
J
K
L
M
N
O
P
GFRA1+
ZBTB16+
KIT+
% EGFP+ Germ Cells
Days in Culture

Fig. S9. Marker analysis of germ cell fates in testes used for FACS. (**A-O**) Immunostaining was done to detect fate markers (ZBTB16 = undifferentiated spermatogonia, KIT = differentiating spermatogonia, SYCP3 = spermatocytes). A few testes from each synchronized litter were saved for immunostaining (shown here), but most of the testes were used for single cell sorting. The age of the collected testis is shown to the left of the panel. Identity of the fate markers are shown at the top of each column (with colors). Note, as expected, KIT is also detectable in a subset of TRA98- /EGFP somatic peritubular myoid and interstitial cells (examples indicated by white arrows). (**P**) Percentages of EGFP+ germ cells expressing each fate marker (GFRA1, not shown, ZBTB16, and KIT). Scale bar (in **A**) = 50 μm. Each representative immunostaining was repeated twice (n=2). Germ cells from over 100 tubules were quantified for each biological replicate. Graphs represent mean +/- standard deviation (s.d.) and error bars represent one s.d.

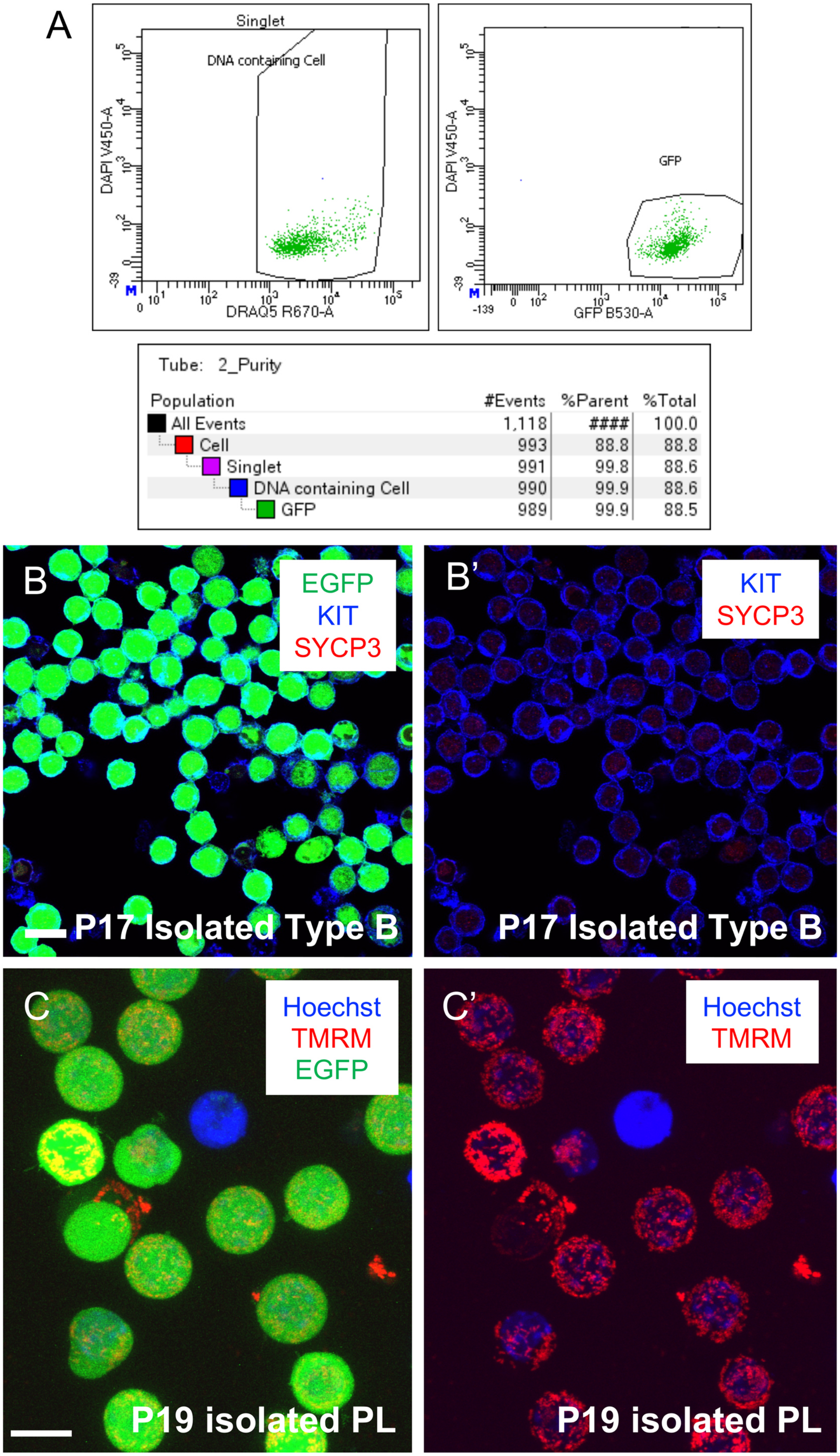

Development • Supplementary information

Fig. S10. Multiparametric validation of FACS results. (**A**) Following FACS (described in Fig. 5L), an aliquot of sorted EGFP+ cells was re-run on the flow cytometer to assess purity of EGFP + cells. (**B-B'**) Immunostaining for protein markers of germ cell fate was done to verify the identity of sorted and plated EGFP+ germ cells. Cells were stained with KIT (blue) and SYCP3 (red). (**C-C'**) A representative image of sorted and plated germ cells from a separate sort to validate the viability of P19 sorted EGFP+ preleptotene spermatocytes. Cells were stained with TMRM (red) and Hoechst (blue). Scale bar = 10 µm. Each representative immunostaining was repeated three times (n=3).

Table S1. Proteomics Data. (**A**) Exported results from Proteome Discoverer 2.2. (**B**) Analyzed results.

Click here to download Table S1

Development • Supplementary information
